# Survival Profile and Prognostic Factors for Appendiceal Mixed Neuroendocrine Non-neuroendocrine Neoplasms: A SEER Population-Based Study

**DOI:** 10.3389/fonc.2020.01660

**Published:** 2020-08-12

**Authors:** Mengzhen Zheng, Tong Li, Yan Li, Tengfei Zhang, Lianfeng Zhang, Wang Ma, Lin Zhou

**Affiliations:** ^1^Department of Gastroenterology, The First Affiliated Hospital of Zhengzhou University, Zhengzhou, China; ^2^Genetic and Prenatal Diagnosis Center, The First Affiliated Hospital of Zhengzhou University, Zhengzhou, China; ^3^Xinxiang Medical University, Xinxiang, China; ^4^Department of Oncology, The First Affiliated Hospital of Zhengzhou University, Zhengzhou, China

**Keywords:** MiNEN, neuroendocrine neoplasms, prognosis, survival, SEER

## Abstract

**Introduction:**

Mixed neuroendocrine non-neuroendocrine neoplasm (MiNEN) is a rare form of neuroendocrine neoplasms (NENs). The purpose of this study was to investigate the characteristics and survival profile of appendiceal MiNENs, with a view of providing robust clinical features of this rare disease.

**Methods:**

Patients were selected from the Surveillance, Epidemiology, and End Results database (2004–2016). The prognosis of MiNEN (*n* = 315) was compared with other histological subtypes including neuroendocrine tumor (NETs) (*n* = 1734), neuroendocrine carcinoma (NECs) (*n* = 375), goblet cell carcinoid (GCC) (*n* = 968), signet ring cell carcinoma (*n* = 463), mucinous adenocarcinoma (MAC) (*n* = 2355), and non-mucinous adenocarcinoma (NMAC) (*n* = 1187) in the appendix. Age-adjusted incidence was calculated using Joinpoint regression. The Cox proportional hazards model and the Fine–Gray competing risk model were used to perform overall survival (OS) and cancer-specific mortality (CSM) analyses, respectively.

**Results:**

The age-adjusted incidence of MiNENs increased from 0.01/100,000 person-years in 2004 to 0.07/100,000 person-years in 2016. The 3-, 5-, and 10-year OS rates for MiNENs were 69.5, 57.4, and 43.7%, respectively, and the corresponding CSM rates were 23.1, 36.4, and 45.1%, respectively. Multivariate analysis revealed that the prognosis of MiNENs was worse than that of NETs, NECs, GCC, and MAC but better than that of NMAC and signet ring cell carcinoma. Tumor extension was the only independent factor influencing the prognosis of MiNENs, but tumor size, grade, and surgical approaches were not. Moreover, when compared with local excision or appendectomy, extensive surgery such as hemicolectomy or colectomy did not prolong the survival of individuals with MiNENs.

**Conclusion:**

MiNEN is a rare but aggressive tumor with a poor prognosis differing from NENs, GCC and adenocarcinomas. To improve the prognosis of the disease, early diagnosis and comprehensive evaluation are necessary.

## Introduction

Mixed neuroendocrine non-neuroendocrine neoplasm (MiNEN) is a rare histological subtype of neuroendocrine neoplasms (NENs). They are hybrid tumors comprising the neuroendocrine and non-neuroendocrine component with each component accounting for at least 30% of the tumor. The first hybrid gastrointestinal tumor was reported in 1924 by Cordier (1). Since then, many inconsistent terms have been used to define these mixed neoplasms, such as goblet cell carcinoid (GCC), collision tumors, adenocarcinoid, composite tumors, and mixed endocrine-exocrine tumor. In 2010, the mixed neoplasms were named as “mixed adeno-neuroendocrine carcinoma (MANEC)” by the World Health Organization (WHO) (2). However, the usage of “MANEC” leads to confusion and misunderstanding, particularly when diagnosing the disease because the spectrum of mixed neoplasms is not merely confined to adenocarcinoma and neuroendocrine carcinoma (NECs) but also low-grade malignancy. For this reason, the term “MiNEN” was recommend to better cover the heterogeneous spectrum of different components (3). Over time, “MiNEN” was eventually made official by the WHO in 2017 (4).

Based on published literature, MiNENs have been reported in almost all digestive tracts including the esophagus, stomach, small bowel, colon, appendix, and rectum as well as other organs (5). Recently, Frizziero et al. (6) found that the majority of the gastro-entero-pancreatic MiNENs occurred in the appendix (60.3%). However, most published studies are case reports and retrospective series with limited sample sizes. Although histological subtypes have been found to be important prognostic factors for appendiceal neoplasms, MiNENs were not defined in the previous study (7). Therefore, the prognosis profile of appendiceal MiNENs is not clear, particularly when compared with other neoplasms occurring in the appendix. Furthermore, optimal treatment strategies for appendiceal MiNENs remain unknown. Generally, the optimal therapeutic management should be informed by the most aggressive component. MiNENs with a poorly differentiated neuroendocrine component should be managed as though they are pure NECs. Conversely, MiNENs with dormant adenocarcinoma component should be treated as adenocarcinomas (8). With regard to MiNENs in the appendix, National Comprehensive Cancer Network (NCCN) guidelines recommend that these tumors should be managed in similar manner as colon cancer (9).

In this study, we focused on the clinical characteristics of appendiceal MiNENs. We compared these characteristics with clinical features for other histological types of appendiceal neoplasms, with a view of providing additional attributes on biological behavior, prognostic features, management and treatment of this rare disease.

## Materials and Methods

### Data Source and Population Selection

This study relied on the “SEER 18 registries” which approximately represent 28% of the United States population. Data for patients in the SEER database stored between 2004 and 2016 was extracted using SEER^∗^Stat software (version 8.3.6, National Center Institute). All the patients diagnosed with appendiceal neoplasms based on the International Classification of Disease for Oncology, third edition (ICD-O-3) primary site code (C18.1, Appendix) were included in this study. The histological subtypes were classified into MiNENs, neuroendocrine tumors (NETs), NECs, GCC, mucinous adenocarcinoma (MAC), non-mucinous adenocarcinoma (NMAC) and signet ring cell carcinoma (SRCC) using identifiable histology codes (Supplementary Table S1). We only included patients positively diagnosed with histology tests. Patients with secondary tumors or other underlying complications or with incomplete data were also excluded from the study. Demographic and clinicopathological characteristics, surgical information and survival time were retrieved for further analyses. Tumor extension was classified into localized (confined to the primary organ), regional (invaded beyond primary organ or involved regional lymph nodes but no distant metastasis) or distant stage (with distant metastasis) based on SEER Combined Summary Stage. The primary endpoints in this study were overall survival (OS) and cancer-specific mortality (CSM). OS was defined as the survival time from diagnosis to death from any causes and CSM was defined as the survival time from diagnosis to death related to tumor.

## Statistical Analysis

The incidence rates for MiNENs were calculated using SEER^∗^Stat software, expressed per 100,000 person-years, and age-adjusted to the year 2000 US standard population. The annual percentage changes (APCs) at 95% confidence interval (CI) of incidence were calculated using the Joinpoint regression software (Version 4.6.0.0, National Cancer Institute). Optimal model was selected using the same software and the significance of APCs evaluated based on *t*-tests.

Demographics and characteristics for different histological subtypes among appendiceal neoplasms were summarized into categorical variables (frequency and percentages) and compared using Chi-square test (or Fisher’s exact test). Skewed continuous variables such as age at diagnosis were analyzed using the Kruskal–Wallis test. The OS curves were drawn using the Kaplan–Meier method and assessed using the log-rank test. Univariate and multivariate Cox proportional hazards models were derived to compare differences in OS among appendiceal neoplasms and to identify independent factors for the prognosis of MiNEN. The hazard ratios (HR) at 95% CI for corresponding risks of all-cause death were also calculated. Furthermore, the Fine–Gray model was derived to perform competing risk analyses by modeling sub-distribution hazard ratios (sHR) to assess CSM, taking into account the competing risk of death from other causes (10). All the analyses were performed using Stata version 15.0 (StataCorp LCC., 4905 Lakeway Drive, College Station, TX, United States) and R 3.5.2 (R Foundation for Statistical Computing, Vienna, Austria). All tests deemed statistically significant were two-sided at *P* < 0.05.

## Results

### Incidence Trends

A total of 401 MiNEN patients reported in SEER 18 registries between 2004 and 2016 fulfilled the inclusion criteria. The age-adjusted incidence (AAI) for the MiNENs increased from 0.01/100,000 person-years in 2004 to 0.07/100,000 person-years in 2016, with an APC of 13.8% (95% CI: 10.0–17.8%, *P* < 0.001). The increasing trends for AAI were observed in both sexes. Males had an APCs of 12.24% (95% CI: 7.2–17.5%, *P* < 0.001) whereas females had 13.81% (95% CI: 8.2–19.7%, *P* < 0.001).

### Demographics and Characteristics for MiNENs

After excluding patients with incomplete follow-up data, 315 MiNEN patients were finally cleared for further analyses. The demographics for the patients are summarized in Table 1. Patients diagnosed with appendiceal NETs (*n* = 1734), NECs (*n* = 375), GCC (*n* = 968), SRCC (*n* = 463), MAC (*n* = 2355), and NMAC (*n* = 1187) were also included in the study for comparisons. Notably, MiNENs are currently one of the rarest histological subtypes among appendiceal neoplasms. The median age of patients diagnosed with MiNENs was 57 years (10–89 years), significantly higher than the median age for those diagnosed with NETs (median age 35 years) and NECs (median age 39 years). In terms of sex, 49.8% were females whereas 50.2% were males. The majority of the MiNEN patients were white (86.7%). Black people accounted for 8.6% of the patients whereas the rest, 4.8%, were from other races.

**TABLE 1:** Demographic and characteristics of appendiceal neoplasms by histological subtypes in SEER database (2004–2016).

Characteristics	MiNENs	NETs	NECs	GCC	SRCC	MAC	NMAC	*P*-value
	*n* = 315 (%)	*n* = 1734 (%)	*n* = 375 (%)	*n* = 968 (%)	*n* = 463 (%)	*n* = 2355 (%)	*n* = 1187 (%)	
**Year at diagnosis**								<0.001
2004–2009	72 (22.9)	155 (8.9)	37 (9.9)	336 (37.8)	167 (36.1)	896 (38.0)	419 (35.3)	
2010–2016	243 (77.1)	1579 (91.1)	338 (90.1)	602 (62.2)	296 (63.9)	1459 (62.0)	768 (64.7)	
**Age at diagnosis**								<0.001
Median (range)	57(10−89)	35(4−92)	39(7−94)	54(8−91)	57(27−94)	58(12−95)	61(19−97)	
≤54	130 (41.3)	1394 (80.4)	281 (74.9)	490 (50.6)	200 (43.2)	972 (41.3)	397 (33.4)	
>54	185 (58.7)	340 (19.6)	94 (25.1)	478 (49.4)	263 (56.8)	1383 (58.7)	790 (66.6)	
**Gender**								<0.001
Female	157 (49.8)	1056 (60.9)	242 (64.5)	474 (49.0)	296 (63.9)	1320 (56.1)	570 (48.0)	
Male	158 (50.2)	678 (39.1)	133 (35.5)	494 (51.0)	167 (36.1)	1035 (43.9)	617 (52.0)	
**Race**								<0.001
White	273 (86.7)	1514 (87.3)	315 (84.0)	825 (85.2)	381 (82.3)	1901 (80.7)	939 (79.1)	
Black	27 (8.6)	116 (6.7)	30 (8.0)	91 (9.4)	46 (9.9)	216 (9.2)	157 (13.2)	
Other^a^	15 (4.8)	104 (6.0)	30 (8.0)	52 (5.4)	36 (7.8)	238 (10.1)	91 (7.7)	
Tumor size*								<0.001
<2 cm	39 (22.4)	1021 (87.6)	220 (71.9)	291 (47.6)	31 (12.3)	182 (15.3)	191 (27.1)	
2–4 cm	62 (35.6)	113 (9.7)	60 (19.6)	169 (27.7)	86 (34.1)	330 (27.7)	237 (33.6)	
≥4 cm	73 (42.0)	32 (2.7)	26 (8.5)	151 (24.7)	135 (53.6)	680 (57.0)	278 (39.4)	
Unknown	141	568	69	357	211	1163	481	
**Tumor extension**								<0.001
Localized	87 (27.6)	1421 (81.9)	262 (69.9)	544 (56.2)	53 (11.4)	437 (18.5)	364 (30.7)	
Regional	122 (38.7)	287 (16.6)	83 (22.1)	306 (31.6)	102 (22.0)	529 (22.5)	428 (36.1)	
Distant	106 (33.7)	26 (1.5)	30 (8.0)	118 (12.2)	308 (66.5)	1389 (59.0)	395 (33.3)	
Grade*								<0.001
I	30 (14.8)	1203 (89.9)	272 (79.8)	135 (40.1)	9 (2.8)	856 (46.0)	160 (15.6)	
II	39 (19.2)	126 (9.4)	48 (14.1)	127 (37.7)	24 (7.4)	762 (40.9)	571 (55.7)	
III or IV	134 (66.0)	9 (0.7)	21 (6.2)	75 (22.3)	292 (89.8)	243 (13.1)	295 (28.8)	
Unknown	112	396	34	631	138	494	161	
**Surgery**								<0.001
Hemicolectomy or more	196 (62.2)	316 (18.2)	117 (31.2)	484 (50.0)	256 (55.3)	1258 (53.4)	656 (55.3)	
Less than hemicolectomy	103 (32.7)	1336 (77.0)	231 (61.6)	436 (45.0)	137 (29.6)	774 (32.9)	400 (33.7)	
Other^b^	16 (5.1)	82 (4.7)	27 (7.2)	48 (5.0)	70 (15.1)	323 (13.7)	131 (11.0)	

*Percentages were calculated based on the non-missing data. ^*a*^American Indian/AK Native, Asian/Pacific Islander, unknown. ^*b*^No surgery or unknown. MiNENs, mixed neuroendocrine non-neuroendocrine neoplasms; NETs, neuroendocrine tumors; NECs, neuroendocrine carcinomas; GCC, goblet cell carcinoid; MAC, mucinous adenocarcinoma; NMAC, non-mucinous adenocarcinoma; SRCC, signet ring cell carcinoma.

Clinical characteristics among different histological subtypes were compared based on the non-missing data such as tumor size and grade. The majority of MiNEN tumors were larger than 4 cm (42.0%), a significantly higher proportion than NETs (2.7%), NECs (8.5%) and GCC (24.7%) but lower than SRCC (53.6%) and MAC (57.0%). With regard to tumor extension, 27.6, 38.7, and 33.7% of MiNEN patients were diagnosed with tumors at localized, regional and distant stage, respectively. In contrast, the majority of NETs, NECs and GCC patients had localized tumors, which respectively accounted for 81.9, 69.9, and 56.2% of the total tumor stages. For SRCC and MAC, the majority of patients were diagnosed with distant stage tumors, respectively accounting for 66.5 and 59.0% of the total cases. In addition, MiNENs presented the highest proportion of tumors at grade III or IV (66.0%), higher than other histological subtypes except SRCC (89.8%).

### Survival Analysis

**FIGURE 1:**
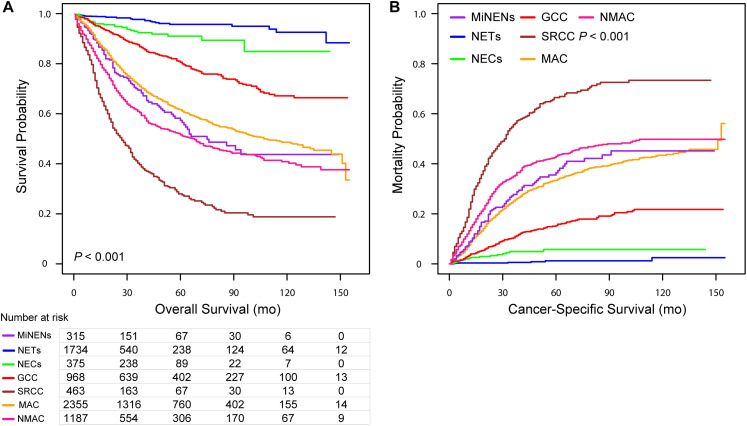
Kaplan–Meier curve for overall survival **(A)** and cumulative incidence curves for cancer-specific mortality **(B)** for patients with appendiceal neoplasms stratified along histological subtypes.

The 3-, 5-, and 10-year OS rates for MiNENs were 69.5, 57.4, and 43.7%, respectively, and the 3-, 5-, and 10-year CSM rates were 23.1, 36.4, and 45.1%, respectively (Table 2). In addition, the respective K-M curves and cumulative incidence curves for OS and CSM are shown in Figure 1. Univariate analysis revealed that the MiNEN patients had a relatively poor OS compared to NETs, NECs, and GCCs (*P* < 0.001) but a better OS compared to SRCC (HR = 2.27, 95% CI: 1.81–2.84, *P* < 0.001). However, there was no significant difference in OS between MiNENs and MAC (HR = 0.87, 95% CI: 0.71–1.07, *P* = 0.194) or NMAC (HR = 1.22, 95% CI: 0.99–1.51, *P* = 0.064) (Supplementary Table S2). We also compared the CSM among patients with different histological subtypes using competing risk model. The results were consistent with the above observations from OS analysis (Supplementary Table S3).

**TABLE 2:** Overall survival and cancer-specific mortality rates of appendiceal neoplasms by histological subtypes.

Histology subtypes	Overall survival			Cancer-specific mortality		
	3-year, %	5-year, %	10-year, %	3-year, %	5-year, %	10-year, %
MiNENs	69.5	57.4	43.7	23.1	36.4	45.1
NETs	97.7	95.7	92.6	0.4	1.2	2.5
NECs	92.4	91	84.9	4.1	5.7	5.7
GCC	87.6	80.6	67.1	9.2	15.2	21.7
SRCC	40.3	27.9	18.8	49.1	66.5	73.3
MAC	72.3	61.6	48.6	21.8	33.3	43.4
NMAC	61.6	51.6	40.8	32.7	43	49.8

*MiNENs, mixed neuroendocrine non-neuroendocrine neoplasms; NETs, neuroendocrine tumors; NECs, neuroendocrine carcinomas; GCC, goblet cell carcinoid, MAC, mucinous adenocarcinoma; NMAC, non-mucinous adenocarcinoma; SRCC, signet ring cell carcinoma.*

**FIGURE 2:**
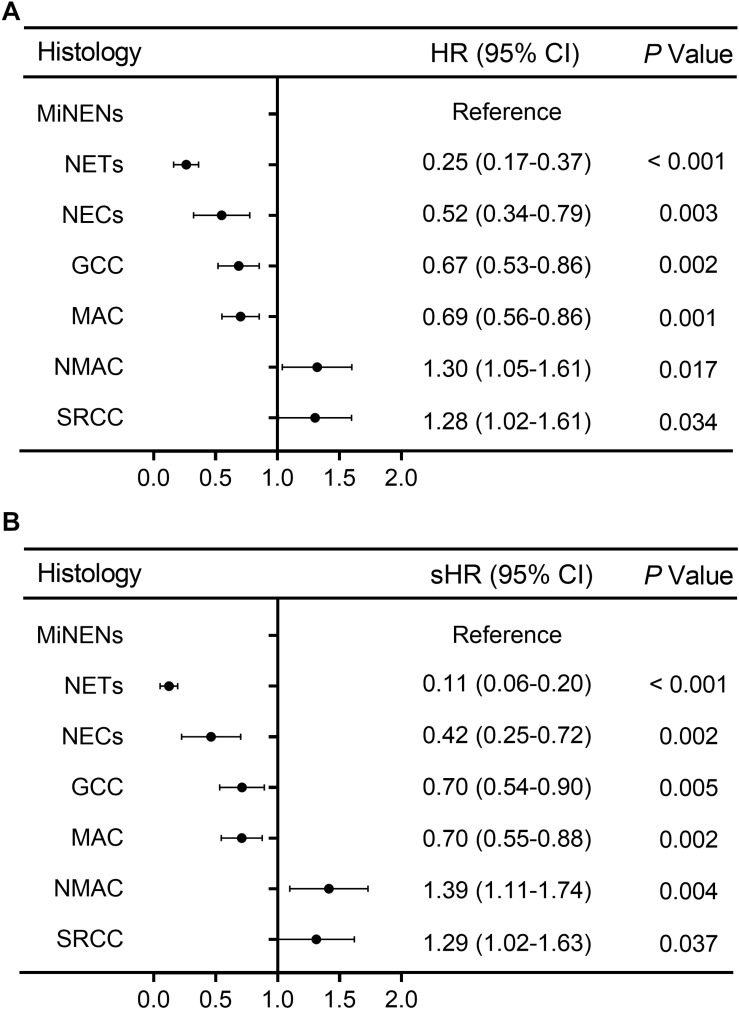
Forest plot for adjusted multivariate analyses along histological subtypes [adjusted HR and sHR were calculated by adding year at diagnosis, age at diagnosis, race, tumor size, extension, grade, and type of surgery into the Cox proportional hazards model **(A)** and competing risks regression model **(B)**, respectively].

Eight clinicopathologic factors significantly associated with survival outcomes in univariate analysis were included in the multivariate analysis. After adjusting for the year at diagnosis, patients’ age, race, tumor size, extension, grade, and type of surgery, histological subtypes were an independent predictive factor related to OS and CSM for appendiceal neoplasms in Cox proportional hazards model and competing risk model (Supplementary Tables S2, S3), respectively. The HRs and sHR are shown in Figure 2, with MiNENs as reference. Patients with NETs, NECs, GCC, and MAC had a better OS but a lower CSM than those with MiNENs. Moreover, patients with SRCC had a higher risk of all-cause death (HR = 1.28, 95% CI: 1.02–1.61, *P* = 0.034) and cancer-specific death (sHR = 1.29, 95% CI: 1.02–1.63, *P* = 0.037) compared to MiNENs. Contrary to the above findings in univariate analyses, multivariate OS and CSM analyses revealed that individuals with NMAC had an increased risk of death compared with patients with MiNENs (HR = 1.30, 95% CI: 1.05–1.61, *P* = 0.017 and sHR = 1.39, 95% CI: 1.11–1.74, *P* = 0.004, respectively).

### Prognostic Factors of Appendiceal MiNENs

**FIGURE 3:**
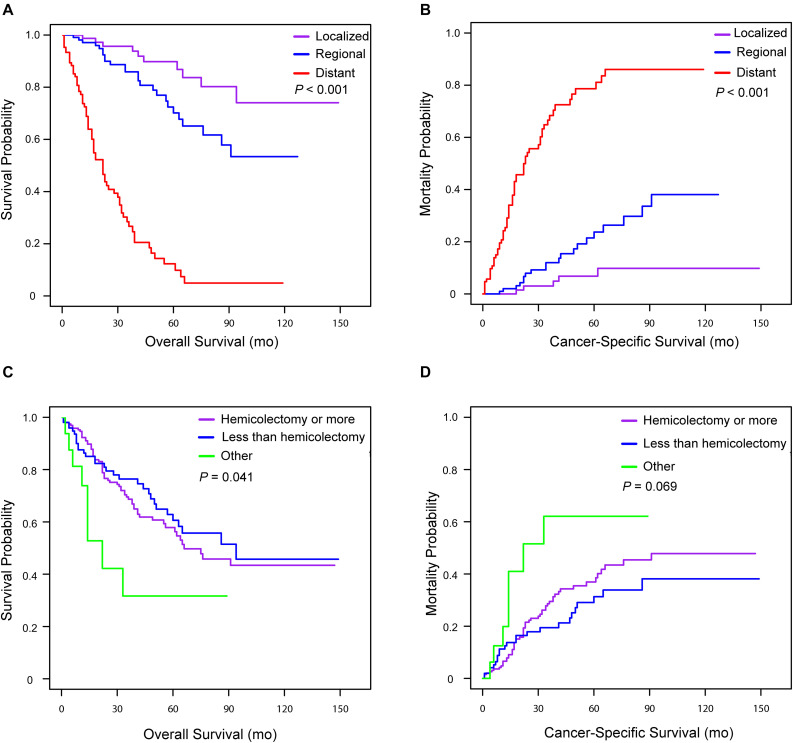
Kaplan–Meier curve for overall survival and cumulative incidence curves for cancer-specific mortality for patients with appendiceal MiNENs stratified by tumor extension **(A,B)** and surgery types **(C,D)**.

**FIGURE 4:**
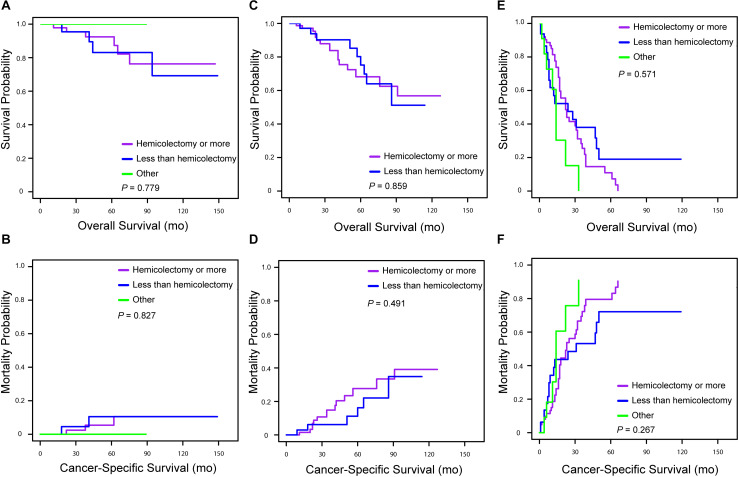
Kaplan–Meier curve for overall survival and cumulative incidence curves for cancer-specific mortality for patients with appendiceal MiNENs stratified by surgery types in tumor extension subgroups [**(A,B)** patients with localized stage; **(C,D)** patients with regional stage; **(E,F)** patients with distant stage].

Univariate and multivariate analyses were also performed to identify factors associated with survival outcomes in MiNEN patients (Tables 3, 4). Patients who were older than 57 years had an increased risk of all-cause death compared to younger patients (HR = 1.60, 95% CI: 1.03–2.47, *P* = 0.035). However, there was no statistical difference in CSM between two groups in patients aged ≤57 and aged >57 (HR = 1.26, 95% CI: 0.78–2.01, *P* = 0.345). Tumor extension was the only independent prognostic factor associated with OS and CSM, whereas tumor size, grade and surgery types were not. To verify the prognostic effect of tumor extension, the multivariate analyses were also performed through three-steps adjustment by building model 1, model 2, and model 3, respectively (Supplementary Tables S4, S5). The results were confirmed that tumor extension was a very reliable predictive factor for the prognosis of MiNENs. Furthermore, patients with localized stage MiNEN had significantly better survival than those with regional and distant stage tumors (Figures 3A,B). The 5-year OS rates were 89.8, 70.2, and 12.3% for localized, regional and distant stage MiNEN, respectively. On the other hand, the 5-year CSM rates were 6.8, 23.7, and 78.6% for localized, regional and distant stage of the disease, respectively. Notably, patients with MiNENs were more likely to undergo surgery. When compared with local excision or appendectomy, hemicolectomy or more extensive surgery did not prolong the OS (HR = 0.89, 95% CI: 0.58–1.35, *P* = 0.576) or lower the CSM (sHR = 0.79, 95% CI: 0.49–1.27, *P* = 0.333) for MiNEN patients (Figures 3C,D), even under subgroups stratified by tumor extension (Figure 4).

**TABLE 3:** Univariate and multivariate Cox proportional hazards analysis of OS in MiNENs patients (*N* = 315).

Characteristics	Univariate analysis		Multivariate analysis	
	HR (95% CI)	*P*-value	HR (95% CI)	*P*-value
**Year at diagnosis**				
2004–2009	Reference	–	Reference	–
2010–2016	1.06 (0.69–1.62)	0.798	0.90 (0.56–1.46)	0.674
**Age at diagnosis**				
≤57	Reference	–	Reference	–
>57	1.71 (1.16–2.53)	0.007	1.60 (1.03–2.47)	0.035
**Gender**				
Female	Reference	–		
Male	0.70 (0.47–1.03)	0.068		
**Race**				
White	Reference	–		
Black	1.21 (0.63–2.32)	0.569		
Other^a^	0.37 (0.09–1.52)	0.169		
**Tumor extension**				
Localized	Reference	–	Reference	–
Regional	2.26 (1.09–4.72)	0.029	2.33 (1.10–4.94)	0.028
Distant	16.42 (8.31–32.42)	<0.001	13.79 (6.72–28.28)	<0.001
**Tumor size**				
<2 cm	Reference	–	Reference	–
2–4 cm	4.26 (1.64–11.08)	0.003	1.91 (0.68–5.34)	0.217
≥4 cm	4.46 (1.72–11.56)	0.002	1.78 (0.65–4.88)	0.263
Unknown	3.48 (1.38–8.82)	0.008	1.88 (0.71–4.95)	0.204
**Grade**				
I	Reference	–	Reference	–
II	0.92 (0.23–3.68)	0.907	0.75 (0.18–3.03)	0.681
III and IV	4.89 (1.77–13.51)	0.002	2.13 (0.74–6.11)	0.160
Unknown	2.98 (1.07–8.31)	0.037	1.48 (0.50–4.35)	0.479
**Surgery**				
Hemicolectomy or more	Reference	–		
Less than hemicolectomy	0.89 (0.58–1.35)	0.576		
Other^b^	2.31 (1.11–4.83)	0.026		

*^*a*^American Indian/AK Native, Asian/Pacific Islander, unknown. ^*b*^No surgery or unknown.*

**TABLE 4:** Univariate and multivariate competing risk analysis of CSM in MiNENs patients (*N* = 315).

Characteristics	Univariate analysis		Multivariate analysis	
	HR (95% CI)	*P*-value	HR (95% CI)	*P*-value
**Year at diagnosis**				
2004–2009	Reference	–	Reference	–
2010–2016	0.94 (0.60–1.49)	0.793	0.80 (0.47–1.35)	0.395
**Age at diagnosis**				
≤57	Reference	–	Reference	–
>57	1.42 (0.93–2.19)	0.106	1.26 (0.78–2.01)	0.345
**Gender**				
Female	Reference	–		
Male	0.71 (0.47–1.07)	0.102		
**Race**				
White	Reference	–		
Black	1.31 (0.66–2.62)	0.430		
Other^a^	0.22 (0.03–1.66)	0.239		
**Tumor extension**				
Localized	Reference	–	Reference	–
Regional	3.46 (1.31–9.11)	0.012	3.17 (1.15–8.77)	0.026
Distant	23.87 (9.60–59.43)	<0.001	19.15 (7.42–49.39)	<0.001
**Tumor size**				
<2 cm	Reference	–	Reference	–
2–4 cm	5.60 (1.63–19.27)	0.006	2.28 (0.53–9.91)	0.271
≥ 4 cm	6.42 (1.89–21.82)	0.003	2.51 (0.58–10.87)	0.216
Unknown	4.54 (1.35–15.27)	0.014	2.32 (0.57–9.43)	0.238
**Grade**				
I	Reference	–	Reference	–
II	0.68 (0.16–2.94)	0.606	0.52 (0.13–2.11)	0.358
III and IV	3.84 (1.45–10.09)	0.006	1.30 (0.49–3.43)	0.596
Unknown	2.54 (0.95–6.79)	0.064	1.20 (0.45–3.17)	0.714
**Surgery**				
Hemicolectomy or more	Reference	–		
Less than hemicolectomy	0.79 (0.49–1.27)	0.333		
Other^b^	2.11 (0.90–4.94)	0.085		

*^*a*^American Indian/AK Native, Asian/Pacific Islander, unknown. ^*b*^No surgery or unknown.*

## Discussion

The aim of our study was to investigate the clinical characteristics of appendiceal MiNENs using the SEER database. Appendiceal MiNEN is a rare form of NENs, accounting for only 5% (401/7483) of the appendiceal neoplasms in our study. The AAI for MiNEN increased by sevenfold from approximately 0.01/100,000 person-years in 2004 to 0.07/100,000 person-years in 2016. The increase may be attributed to improved clinical recognition and better diagnostic technologies over the years. This study demonstrated that MiNENs were moderately aggressive compared with NENs and adenocarcinoma. Moreover, tumor extension was shown to be the only prognostic factor associated with OS and CSM in MiNEN patients.

The prognosis of well differentiated NETs (G1 and G2) located in the appendix is satisfactory. Previous studies have reported 5-year OS rates for NETs to be between 88 and 96% (11). However, the comparative survival outcome between MiNENs and NECs is still debatable. Based on our study, survival analysis revealed that the prognosis of patients with MiNENs was significantly worse than that of patients with NETs or NECs. Notably, most patients with MiNENs are older, and in most cases, diagnosed with poorly differentiated disease (Table 1), which may explain the poor prognosis for individuals diagnosed. Contrary to our results, La Rosa et al. (12, 13) found that patients with gastric MiNENs presented a better prognosis than patients with NECs, but the prognosis of patients with colorectal MiNENs and pure NECs was indifferent. The difference in survival between individuals with MiNENs and NECs may be site-related (14).

Both MiNENs and GCC are believed to develop from crypt base stem cells by abnormal differentiation of both neuroendocrine and glandular cells. Historically, MiNENs and GCC were categorized into the same pathologic type and many studies failed to distinguish between them (15, 16). The WHO classification differentiated MiNENs from GCC as a distinct clinical entity, streamlining the previous classification done in 2008 by Tang et al. (17). Our findings supported this subclassification because patients with appendiceal MiNENs had a higher risk of all-cause death and cancer-specific death than those with GCC. Likewise, Brathwaite et al. (18) found significant difference in median OS between appendiceal MiNENs and GCC (6.5 years vs 13.8 years, *P* < 0.0001). Moreover, patients with MiNENs were more likely to be diagnosed with III or IV stage of the disease than those with GCC, which was also consistent with our findings. Thus, adding further evidence that the appendiceal MiNENs might be more aggressive than GCC.

A previous study performed by Watanabe et al. (19) revealed that colorectal MiNENs has a worse 5-year OS rate of 69.0% against 82.0% for adenocarcinomas (*P* = 0.048). However, the survival differences for appendiceal lesions remain unclear. In this study, appendiceal adenocarcinomas were further subdivided into three histological types: mucinous, non-mucinous, and signet ring cell type. It was found that the survival rate for patients with MiNENs was significantly worse than those with MAC but better than those with NMAC and SRCC. With regard to genetic level, MiNENs and conventional adenocarcinomas share a broadly similar copy number aberration profile (20). Moreover, genetic profiling for both neuroendocrine and adenocarcinomas components in colorectal MiNENs demonstrated that both components share a repertoire of carcinogenetic genes (21). To a large extent, this supports the hypothesis that the two MiNENs components share a monoclonal origin during tumorigenesis (6). Additionally, the high proportion of MiNENs diagnosed with an advanced stage or poor differentiation may suggest that the biological behavior of the disease is similar to that of adenocarcinoma rather than pure NECs or GCC.

To our knowledge, factors affecting the prognosis of appendiceal MiNENs have not yet been reported. Multivariate analyses demonstrated that tumor extension was the only independent factor associated with the OS and CSM of patients with appendiceal MiNENs. Patients with distant MiNENs had the worst prognosis, with 5-year OS and CSM rates of 12.3 and 78.6%, respectively. One study found that metastatic gastrointestinal MiNENs had a higher incidence than localized disease (22). In our study, the proportion of patients with an advanced stage MiNENs was approximately 33% and this proportion was even higher in other studies (23, 24). Therefore, early identification and treatment are integral factors that can improve the survival of patients.

There are no clear surgical guidelines and uniform treatment standards for MiNENs due to their rarity and heterogeneity. For localized or regional disease, a curative-intent resection, with or without lymphadenectomy, should be performed whenever feasible (25). As the high risk of recurrence after R0 resection, some studies suggested that adjuvant treatment like chemotherapy prolonged the progression-free survival and OS of patients, though most of them were case studies (26). In our study, more than 90% of patients, even with advanced diseases, had their MiNENs surgical excised. Notably, extensive surgery such as hemicolectomy did not increase the survival rate of patients (Figure 4). When compared with local excision or appendectomy, hemicolectomy or total colectomy increase the risk of postoperative complications, which may negatively impact on both the quality of life and survival (27, 28). For metastatic MiNENs, the histologic aspect of metastatic component should be fully considered when choosing an appropriate treatment strategy (29). Generally, it depends on one of the most predominant or aggressive component in metastases. However, the metastatic lesions are usually occupied by a single component (most frequently the poorly differentiated NECs). Thus, biopsy of metastases before planning treatment strategy is necessary and the treatment should target the unique component responsible for the metastatic spreading rather than based on the characteristics of the primary site (5, 30). Cisplatin and etoposide are recommended as the first-line regimen when the metastatic lesion predominantly comprises NEC component (31, 32). In addition, schemes with carboplatin and etoposide or cisplatin and irinotecan have also been recommended as an adjuvant chemotherapy (9). If the prominent component is adenocarcinoma in metastases, established regimens for “pure” adenocarcinoma are treatment options. Overall, therapeutic decisions should be based on multidisciplinary assessments, taking into consideration the probable implications of the proposed approach.

This study had several limitations. First, given that it was a retrospective study, it was impossible to avoid any selection bias. Second, there is a wide spectrum of possible combinations between neuroendocrine and non-neuroendocrine component (3). Chen et al. (33) found that high proportions of neuroendocrine component (>50%) in MiNENs adversely affected the survival of respective patients, suggesting that the predominant tumor component influences the prognosis of the disease. Elsewhere, the Ki67 proliferative index in NEC component (above or below 55%) was also reported to significantly influence the prognosis of MiNENs (34). However, in our study, the contribution of these variables was not evaluated. Additionally, information on chemotherapy, radiotherapy or targeted therapy was not available in the SEER database. Therefore, these potential prognosis associated factors were not included in the Cox regression and competing risk model for adjusting the bias. This omission might have influenced our findings, which may limit the determination of optimal management. Therefore, further studies need to expound on the role of these factors on the prognosis to provide guidelines for the treatment of MiNENs.

## Conclusion

Despite these limitations, our study demonstrated that appendiceal MiNENs is a distinct disease with a dual histological nature. MiNENs are more aggressive than NENs but similar to adenocarcinoma, implying that a more aggressive treatment strategy should be preferred, but based on extensive multidisciplinary evaluation and consultation. Tumor extension is a very reliable predictive factor for the prognosis of MiNENs, thus it should be considered when selecting the best treatment strategies.

## Data Availability Statement

Publicly available datasets were analyzed in this study. This data can be found here: The Surveillance, Epidemiology, and End Results database (https://seer.cancer.gov/).

## Author Contributions

LZho and WM participated in the conception and design of the study. MZ collected the data. MZ and TL performed the analysis and interpretation of the data. MZ, TL, and TZ participated in the designing and writing of the manuscript. WM, LZho, YL, and LZha revised the manuscript. All authors reviewed and approved the final manuscript.

## Conflict of Interest

The authors declare that the research was conducted in the absence of any commercial or financial relationships that could be construed as a potential conflict of interest.
