# Supplementary material for: Survival Profile and Prognostic Factors for Appendiceal Mixed Neuroendocrine Non-neuroendocrine Neoplasms: A SEER Population-Based Study
Source: Front Oncol. 2020 Aug 12;10:1660. doi: 10.3389/fonc.2020.01660 (PMC7438709; doi:10.3389/fonc.2020.01660)
Supplement: Supplementary file 1 [file Data_Sheet_1.docx]

| **Table S1** Frequency of appendiceal neuroendocrine tumor histologies in SEER database (2004-2016). | | | |
| --- | --- | --- | --- |
| Tumor subtypes | ICD-O-3 Code | Defination | Cases (N=7397) |
| MiNENs | 8244 | Mixed adenoneuroendocrine carcinoma (ICD-O-3 update) | 315 |
| NETs | 8240 | Carcinoid tumor, NOS | 1727 |
|  | 8241 | Enterochromaffin cell carcinoid | 7 |
| NECs | 8013 | Large cell neuroendocrine carcinoma | 6 |
|  | 8246 | Neuroendocrine carcinoma, NOS | 369 |
| GCC | 8243 | Goblet cell carcinoid | 797 |
|  | 8245 | Adenocarcinoid tumor | 171 |
| MAC | 8470 | Mucinous cystadenocarcinoma, NOS | 180 |
|  | 8471 | Papillary mucinous cystadenocarcinoma | 3 |
|  | 8480 | Mucinous adenocarcinoma | 2072 |
|  | 8481 | Mucin-producing adenocarcinoma | 100 |
| NMAC | 8140 | Adenocarcinoma, NOS | 1048 |
|  | 8144 | Adenocarcinoma, intestinal type | 8 |
|  | 8211 | Tubular adenocarcinoma | 1 |
|  | 8255 | Adenocarcinoma with mixed subtypes | 113 |
|  | 8262 | Villous adenocarcinoma | 1 |
|  | 8310 | Clear cell adenocarcinoma, NOS | 0 |
|  | 8440 | Cystadenocarcinoma, NOS | 15 |
|  | 8460 | Papillary serous cystadenocarcinoma | 1 |
| SRCC | 8490 | Signet ring cell carcinoma | 463 |
| Abbreviations: MiNENs: mixed neuroendocrine non-neuroendocrine neoplasms; NETs: neuroendocrine tumors; NECs: neuroendocrine carcinomas; GCC: goblet cell carcinoid, MAC: mucinous adenocarcinoma; NMAC: non-mucinous adenocarcinoma; SRCC: signet ring cell carcinoma. | | | |

| **Table S2** Univariate and multivariate Cox proportional hazards analysis of overall survival in all cohort (N=7397) | | | | | | |
| --- | --- | --- | --- | --- | --- | --- |
| Characteristics | Univariable | |  | Multivariate | |  |
|  | HR (95%CI) | *P* value |  | HR (95%CI) | *P* value |  |
| Year at diagnosis |  |  |  |  |  |  |
| 2004-2009 | Ref. | - |  | Ref. | - |  |
| 2010-2016 | 0.77 (0.70-0.85) | ＜0.001 |  | 0.83 (0.75-0.91) | ＜0.001 |  |
| Age at diagnosis |  |  |  |  |  |  |
| ≤ 54 | Ref. | - |  | Ref. | - |  |
| > 54 | 2.00 (1.83-2.20) | ＜0.001 |  | 1.57 (1.43-1.73) | ＜0.001 |  |
| Gender |  |  |  |  |  |  |
| Female | Ref. | - |  |  |  |  |
| Male | 1.04 (0.95-1.13) | 0.406 |  |  |  |  |
| Race |  |  |  |  |  |  |
| White | Ref. | - |  | Ref. | - |  |
| Black | 1.38 (1.21-1.58) | ＜0.001 |  | 1.44 (1.25-1.64) | ＜0.001 |  |
| Other^a^ | 1.10 (0.93-1.30) | 0.254 |  | 0.98 (0.83-1.16) | 0.818 |  |
| Tumor size |  |  |  |  |  |  |
| ＜2cm | Ref. | - |  | Ref. | - |  |
| 2cm-4cm | 2.81 (2.36-3.34) | ＜0.001 |  | 1.19 (0.99-1.42) | 0.065 |  |
| ≥4cm | 4.32 (3.69-5.06) | ＜0.001 |  | 1.36 (1.15-1.61) | ＜0.001 |  |
| unknown | 3.58 (3.08-4.17) | ＜0.001 |  | 1.21 (1.03-1.43) | 0.022 |  |
| Tumor extension |  |  |  |  |  |  |
| Localized | Ref. | - |  | Ref. | - |  |
| Regional | 2.79 (2.39-3.25) | ＜0.001 |  | 2.09 (1.78-2.44) | ＜0.001 |  |
| Distant | 8.21 (7.17-9.40) | ＜0.001 |  | 5.50 (4.72-6.41) | ＜0.001 |  |
| Grade |  |  |  |  |  |  |
| I | Ref. | - |  | Ref. | - |  |
| II | 2.45 (2.11-2.85) | ＜0.001 |  | 1.64 (1.40-1.91) | ＜0.001 |  |
| III and IV | 6.40 (5.54-7.38) | ＜0.001 |  | 3.00 (2.55-3.52) | ＜0.001 |  |
| Unknown | 2.43 (2.10-2.80) | ＜0.001 |  | 2.08 (1.78-2.42) | ＜0.001 |  |
| Histology subtypes |  |  |  |  |  |  |
| MiNENs | Ref. | - |  | Ref. | - |  |
| NETs | 0.08 (0.05-0.11) | ＜0.001 |  | 0.25 (0.17-0.37) | ＜0.001 |  |
| NECs | 0.19 (0.12-0.27) | ＜0.001 |  | 0.52 (0.34-0.79) | 0.003 |  |
| GCC | 0.42 (0.33-0.53) | ＜0.001 |  | 0.67 (0.53-0.86) | 0.002 |  |
| SRCC | 2.27 (1.81-2.84) | ＜0.001 |  | 1.28 (1.02-1.61) | 0.034 |  |
| MAC | 0.87 (0.71-1.07) | 0.194 |  | 0.69 (0.56-0.86) | 0.001 |  |
| NMAC | 1.22 (0.99-1.51) | 0.064 |  | 1.30 (1.05-1.61) | 0.017 |  |
| Surgery |  |  |  |  |  |  |
| Hemicolectomy or more | Ref. | - |  | Ref. | - |  |
| Less than hemicolectomy | 0.73 (0.66-0.81) | ＜0.001 |  | 1.13 (1.02-1.25) | 0.021 |  |
| Other^b^ | 2.37 (2.10-2.67) | ＜0.001 |  | 1.92 (1.67-2.20) | ＜0.001 |  |
| ^a^ American Indian/AK Native, Asian/Pacific Islander, unknown. | | | | | |  |
| ^b^ No surgery or unknown. | | | | | |  |
| Abbreviations: MiNENs: mixed neuroendocrine non-neuroendocrine neoplasms; NETs: neuroendocrine tumors; NECs: neuroendocrine carcinomas; GCC: goblet cell carcinoid, MAC: mucinous adenocarcinoma; NMAC: non-mucinous adenocarcinoma; SRCC: signet ring cell carcinoma. | | | | | |  |

| **Table S3** Univariate and multivariate Cox proportional hazards analysis of cancer-specific mortality  in all cohort (N=7397). | | | | | | |
| --- | --- | --- | --- | --- | --- | --- |
| Characteristics | Univariable | |  | Multivariate | |  |
|  | sHR (95%CI) | *P* value |  | sHR (95%CI) | *P* value |  |
| Year at diagnosis |  |  |  |  |  |  |
| 2004-2009 | Ref. | - |  | Ref. | - |  |
| 2010-2016 | 0.75 (0.68-0.83) | ＜0.001 |  | 0.80 (0.72-0.88) | ＜0.001 |  |
| Age at diagnosis |  |  |  |  |  |  |
| ≤ 54 | Ref. | - |  | Ref. | - |  |
| > 54 | 1.72 (1.56-1.89) | ＜0.001 |  | 1.30 (1.18-1.44) | ＜0.001 |  |
| Gender |  |  |  |  |  |  |
| Female | Ref. | - |  |  |  |  |
| Male | 0.96 (0.87-1.05) | 0.367 |  |  |  |  |
| Race |  |  |  |  |  |  |
| White | Ref. | - |  | Ref. | - |  |
| Black | 1.33 (1.14-1.54) | ＜0.001 |  | 1.36 (1.17-1.60) | ＜0.001 |  |
| Other^a^ | 1.07 (0.90-1.29) | 0.416 |  | 0.91 (0.76-1.09) | 0.004 |  |
| Tumor size |  |  |  |  |  |  |
| ＜2cm | Ref. | - |  | Ref. | - |  |
| 2cm-4cm | 3.66 (2.98-4.50) | ＜0.001 |  | 1.29 (1.05-1.59) | 0.015 |  |
| ≥4cm | 5.75 (4.75-6.96) | ＜0.001 |  | 1.45 (1.19-1.77) | ＜0.001 |  |
| unknown | 4.90 (4.07-5.90) | ＜0.001 |  | 1.32 (1.09-1.60) | 0.004 |  |
| Tumor extension |  |  |  |  |  |  |
| Localized | Ref. | - |  | Ref. | - |  |
| Regional | 4.60 (3.74-5.64) | ＜0.001 |  | 3.20 (2.59-3.96) | ＜0.001 |  |
| Distant | 15.63 (12.98-18.82) | ＜0.001 |  | 9.78 (7.94-12.05) | ＜0.001 |  |
| Grade |  |  |  |  |  |  |
| I | Ref. | - |  | Ref. | - |  |
| II | 2.58 (2.19-3.05) | ＜0.001 |  | 1.67 (1.40-1.98) | ＜0.001 |  |
| III and IV | 7.33 (6.27-8.58) | ＜0.001 |  | 3.04 (2.54-3.64) | ＜0.001 |  |
| Unknown | 2.69 (2.29-3.16) | ＜0.001 |  | 2.18 (1.84-2.59) | ＜0.001 |  |
| Histology subtypes |  |  |  |  |  |  |
| MiNENs | Ref. | - |  | Ref. | - |  |
| NETs | 0.03 (0.01-0.05) | ＜0.001 |  | 0.11 (0.06-0.20) | ＜0.001 |  |
| NECs | 0.13 (0.08-0.23) | ＜0.001 |  | 0.42 (0.25-0.72) | 0.002 |  |
| GCC | 0.38 (0.29-0.49) | ＜0.001 |  | 0.70 (0.54-0.90) | 0.005 |  |
| SRCC | 2.39 (1.88-3.03) | ＜0.001 |  | 1.29 (1.02-1.63) | 0.037 |  |
| MAC | 0.91 (0.73-1.13) | 0.378 |  | 0.70 (0.55-0.88) | 0.002 |  |
| NMAC | 1.25 (1.00-1.58) | 0.051 |  | 1.39 (1.11-1.74) | 0.004 |  |
| Surgery |  |  |  |  |  |  |
| Hemicolectomy or more | Ref. | - |  | Ref. | - |  |
| Less than hemicolectomy | 0.68 (0.61-0.76) | ＜0.001 |  | 1.10 (0.98-1.23) | 0.097 |  |
| Other^b^ | 2.44 (2.13-2.80) | ＜0.001 |  | 1.78 (1.52-2.08) | ＜0.001 |  |
| ^a^ American Indian/AK Native, Asian/Pacific Islander, unknown. | | | | | |  |
| ^b^ No surgery or unknown. | | | | | |  |
| Abbreviations: MiNENs: mixed neuroendocrine non-neuroendocrine neoplasms; NETs: neuroendocrine tumors; NECs: neuroendocrine carcinomas; GCC: goblet cell carcinoid, MAC: mucinous adenocarcinoma; NMAC: non-mucinous adenocarcinoma; SRCC: signet ring cell carcinoma. | | | | | |  |

| **TABLE S4** Multivariate Cox proportional hazards analysis of OS in MiNENs patients (N=315). | | | | | | | | | |
| --- | --- | --- | --- | --- | --- | --- | --- | --- | --- |
| Characteristics | Multivariate analysis | | | | | | | | |
|  | Model 1 | |  | Model 2 | |  | Model 3 | |  |
|  | HR (95% CI) | *P* value |  | HR (95% CI) | *P* value |  | HR (95% CI) | *P* value |  |
| Year at diagnosis |  |  |  |  |  |  |  |  |  |
| 2004-2009 | Ref. | - |  | Ref. | - |  | Ref. | - |  |
| 2010-2016 | 0.92 (0.59-1.43) | 0.702 |  | 0.92 (0.56-1.53) | 0.761 |  | 0.90 (0.55-1.48) | 0.67 |  |
| Age at diagnosis |  |  |  |  |  |  |  |  |  |
| ≤ 57 | Ref. | - |  | Ref. | - |  | Ref. | - |  |
| > 57 | 1.92 (1.24-2.98) | 0.004 |  | 1.84 (1.18-2.87) | 0.008 |  | 1.83 (1.17-2.86) | 0.008 |  |
| Gender |  |  |  |  |  |  |  |  |  |
| Female | Ref. | - |  | Ref. | - |  | Ref. | - |  |
| Male | 0.71 (0.47-1.08) | 0.111 |  | 0.71 (0.47-1.10) | 0.123 |  | 0.70 (0.45-1.07) | 0.096 |  |
| Race |  |  |  |  |  |  |  |  |  |
| White | Ref. | - |  | Ref. | - |  | Ref. | - |  |
| Black | 1.95 (0.98-3.86) | 0.056 |  | 2.08 (1.04-4.17) | 0.04 |  | 2.15 (1.07-4.32) | 0.032 |  |
| Other^a^ | 0.30 (0.07-1.22) | 0.092 |  | 0.29 (0.07-1.19) | 0.085 |  | 0.27 (0.07-1.13) | 0.072 |  |
| **Tumor extension** |  |  |  |  |  |  |  |  |  |
| **Localized** | **Ref.** | **-** |  | **Ref.** | **-** |  | **Ref.** | **-** |  |
| **Regional** | **2.65 (1.26-5.58)** | **0.01** |  | **2.55 (1.19-5.46)** | **0.016** |  | **2.63 (1.22-5.66)** | **0.014** |  |
| **Distant** | **19.1 (9.47-38.48)** | **＜0.001** |  | **16.3 (7.86-33.76)** | **＜0.001** |  | **15.9 (7.60-33.3)** | **＜0.001** |  |
| Tumor size |  |  |  |  |  |  |  |  |  |
| ＜2cm |  |  |  | Ref. | - |  | Ref. | - |  |
| 2cm-4cm |  |  |  | 1.76 (0.63-4.93) | 0.281 |  | 1.88 (0.66-5.35) | 0.236 |  |
| ≥4cm |  |  |  | 1.56 (0.57-4.26) | 0.391 |  | 1.66 (0.60-4.61) | 0.329 |  |
| unknown |  |  |  | 1.86 (0.71-4.86) | 0.205 |  | 1.84 (0.70-4.87) | 0.22 |  |
| Grade |  |  |  |  |  |  |  |  |  |
| I |  |  |  | Ref. | - |  | Ref. | - |  |
| II |  |  |  | 0.85 (0.21-3.51) | 0.823 |  | 0.86 (0.21-3.55) | 0.831 |  |
| III and IV |  |  |  | 2.35 (0.81-6.83) | 0.117 |  | 2.48 (0.85-7.26) | 0.096 |  |
| Unknown |  |  |  | 1.82 (0.61-5.43) | 0.283 |  | 1.75 (0.58-5.27) | 0.318 |  |
| Surgery |  |  |  |  |  |  |  |  |  |
| Hemicolectomy or more |  |  |  |  |  |  | Ref. | - |  |
| Less than hemicolectomy |  |  |  |  |  |  | 0.98 (0.63-1.54) | 0.934 |  |
| Other^b^ |  |  |  |  |  |  | 2.48 (1.10-5.59) | 0.028 |  |
| ^a^ American Indian/AK Native, Asian/Pacific Islander, unknown. | | | | | | | | | |
| ^b^ No surgery or unknown. | | | | | | | | | |

| **TABLE S5** Multivariate competing risk analysis of CSM in MiNENs patients (N=315). | | | | | | | | | |
| --- | --- | --- | --- | --- | --- | --- | --- | --- | --- |
| Characteristics | Multivariate analysis | | | | | | | | |
|  | Model 1 | |  | Model 2 | |  | Model 3 | |  |
|  | sHR (95% CI) | *P* value |  | sHR (95% CI) | *P* value |  | sHR (95% CI) | *P* value |  |
| Year at diagnosis |  |  |  |  |  |  |  |  |  |
| 2004-2009 | Ref. | - |  | Ref. | - |  | Ref. | - |  |
| 2010-2016 | 0.71 (0.45-1.13) | 0.146 |  | 0.71 (0.41-1.23) | 0.22 |  | 0.69 (0.40-1.19) | 0.183 |  |
| Age at diagnosis |  |  |  |  |  |  |  |  |  |
| ≤ 57 | Ref. | - |  | Ref. | - |  | Ref. | - |  |
| > 57 | 1.51 (0.95-2.41) | 0.082 |  | 1.43 (0.88-2.31) | 0.146 |  | 1.47 (0.91-2.38) | 0.116 |  |
| Gender |  |  |  |  |  |  |  |  |  |
| Female | Ref. | - |  | Ref. | - |  | Ref. | - |  |
| Male | 0.81 (0.53-1.25) | 0.346 |  | 0.82 (0.52-1.28) | 0.385 |  | 0.80 (0.51-1.24) | 0.318 |  |
| Race |  |  |  |  |  |  |  |  |  |
| White | Ref. | - |  | Ref. | - |  | Ref. | - |  |
| Black | 2.52 (1.31-4.85) | 0.006 |  | 2.41 (1.23-4.71) | 0.01 |  | 2.49 (1.27-4.86) | 0.008 |  |
| Other^a^ | 0.15 (0.02-1.27) | 0.082 |  | 0.14 (0.02-1.23) | 0.076 |  | 0.14 (0.02-1.14) | 0.066 |  |
| **Tumor extension** |  |  |  |  |  |  |  |  |  |
| **Localized** | **Ref.** | **-** |  | **Ref.** | **-** |  | **Ref.** | **-** |  |
| **Regional** | **3.91 (1.42-10.76)** | **0.008** |  | **2.32 (0.49-10.91)** | **0.026** |  | **3.59 (1.18-10.95)** | **0.025** |  |
| **Distant** | **28.69 (10.87-75.70)** | **＜0.001** |  | **22.92 (8.20-64.03)** | **＜0.001** |  | **22.54 (7.94-63.97)** | **＜0.001** |  |
| Tumor size |  |  |  |  |  |  |  |  |  |
| ＜2cm |  |  |  | Ref. | - |  | Ref. | - |  |
| 2cm-4cm |  |  |  | 2.32 (0.49-10.91) | 0.286 |  | 2.18 (0.47-10.09) | 0.319 |  |
| ≥4cm |  |  |  | 2.26 (0.49-10.50) | 0.297 |  | 2.19 (0.47-10.20) | 0.317 |  |
| unknown |  |  |  | 2.28 (0.53-9.92) | 0.27 |  | 2.12 (0.49-9.07) | 0.313 |  |
| Grade |  |  |  |  |  |  |  |  |  |
| I |  |  |  | Ref. | - |  | Ref. | - |  |
| II |  |  |  | 0.63 (0.14-2.71) | 0.531 |  | 0.68 (0.15-3.06) | 0.613 |  |
| III and IV |  |  |  | 1.47 (0.52-4.13) | 0.469 |  | 1.51 (0.53-4.27) | 0.437 |  |
| Unknown |  |  |  | 1.38 (0.50-3.85) | 0.532 |  | 1.36 (0.49-3.78) | 0.561 |  |
| Surgery |  |  |  |  |  |  |  |  |  |
| Hemicolectomy or more |  |  |  |  |  |  | Ref. | - |  |
| Less than hemicolectomy |  |  |  |  |  |  | 0.88 (0.50-1.55) | 0.665 |  |
| Other^b^ |  |  |  |  |  |  | 1.63 (0.69-3.89) | 0.268 |  |
| ^a^ American Indian/AK Native, Asian/Pacific Islander, unknown. | | | | | | | | | |
| ^b^ No surgery or unknown. | | | | | | | | | |

**Code for generating the Fine-Gray competing risk model with R**

library("foreign")

bmt = read.spss("bmt.sav", to.data.frame=TRUE)

attach(bmt)

bmt$subtypes <- factor(bmt$subtypes,

levels = c(1,2,3,4,5,6,7),

labels = c("MiNENs", "NETs", "NECs", "GCC", "SRCC", "MAC", "NMAC")

)

library("splines")

library("survival")

library("cmprsk")

**Generate cumulative incidence function (CIF) and plot of cumulative incidence curve**

uni_crm <- cuminc(Time, CSS, subtypes)

plot(uni_crm$"MiNENs 1"$time, uni_crm$"MiNENs 1"$est, type = "s", lty=1, xlim = c(0,160), ylim = c(0.0,1.0), xlab = "Survival (mo)", ylab = "Mortality probability", lwd = 2, col = "purple", xaxt = "n", yaxt = "n")

lines(uni_crm$"NETs 1"$time, uni_crm$"NETs 1"$est, type="s", col="blue", lty =1,lwd =2)

lines(uni_crm$"NECs 1"$time, uni_crm$"NECs 1"$est, type="s", col="green", lty =1,lwd =2)

lines(uni_crm$"GCC 1"$time, uni_crm$"GCC 1"$est, type="s", col="red", lty =1,lwd =2)

lines(uni_crm$"SRCC 1"$time, uni_crm$"SRCC 1"$est, type="s", col="brown", lty =1,lwd =2)

lines(uni_crm$"MAC 1"$time, uni_crm$"MAC 1"$est, type="s", col="orange", lty =1,lwd =2)

lines(uni_crm$"NMAC 1"$time, uni_crm$"NMAC 1"$est, type="s", col="deeppink", lty =1,lwd =2)

axis(side=1, at=c(0,30,60,90,120,150), labels=c(0,30,60,90,120,150))

axis(side=2, at=c(0,0.2,0.4,0.6,0.8,1.0), labels=c(0,0.2,0.4,0.6,0.8,1.0))

legend("top",c("MiNENs","NETs","NECs","GCC","SRCC","MAC","NMAC"),col=c("purple","blue","green","red","brown","orange","deeppink"), lty=c(1), lwd =2, bty = "n", ncol=3)

**For univariate analysis**

cov_1<-model.matrix(~ subtypes, data= bmt)[,-1]

uni_crr <- crr(bmt$Time, bmt$CSS, cov1= cov_1, failcode=1, cencode=0)

summary(uni_crr)

**For multivariate analysis**

cov_2 <-model.matrix(~ age + race + size + extension + subtypes + surgery, data= bmt)[,-1]

multi_crr <- crr(bmt$Time, bmt$CSS, cov1= cov_2, failcode=1, cencode=0)

summary(multi_crr)
